# Postoperative kinetics of point-of-care C-reactive protein versus total white blood cell count after tibial plateau leveling osteotomy in dogs

**DOI:** 10.14202/vetworld.2026.1785-1795

**Published:** 2026-04-30

**Authors:** Thirawat Sumalai, Sakchai Ruenphet, Supphathat Wutthiwitthayaphong, Rachakris Lertpatarakomol, Tassanee Trairatapiwan

**Affiliations:** 1 Animal Biotechnology, Mahanakorn University of Technology, Bangkok, Thailand; 2 Immunology and Virology, Mahanakorn University of Technology, Bangkok, Thailand

**Keywords:** C-reactive protein, tibial plateau leveling osteotomy, point-of-care CRP analyzer, white blood cell count, acute phase response, postoperative monitoring

## Abstract

**Background and Aim:**

Objective assessment of the postoperative acute phase response following major orthopedic surgery, such as tibial plateau leveling osteotomy (TPLO), is essential for distinguishing uncomplicated recovery from infectious complications. Serum C-reactive protein (CRP) levels typically return to baseline by 14 days postoperatively, whereas total white blood cell (WBC) counts may remain nonspecifically elevated due to physiological stress. This study aimed to prospectively characterize and directly compare the postoperative kinetics of point-of-care CRP and total WBC counts in dogs undergoing TPLO.

**Materials and Methods:**

Thirteen client-owned dogs undergoing standardized TPLO (classified as Level 4 severity) were prospectively enrolled. Venous blood samples were collected preoperatively (Day 0) and on postoperative days 1, 3, 5, 7, 10, and 14. Serum CRP concentrations were measured using a validated point-of-care immunofluorescence assay (SmartDiag®), and total WBC counts were determined concurrently using an automated hematology analyzer. Temporal changes in both biomarkers were analyzed using a repeated-measures one-way analysis of variance with Dunnett’s post hoc test.

**Results:**

Both biomarkers increased significantly from baseline and peaked on postoperative day 1 (p < 0.05). However, their subsequent kinetics diverged markedly. Mean serum CRP concentrations showed a rapid and progressive decline, approaching preoperative baseline levels by postoperative day 7 and remaining stable thereafter. In contrast, mean total WBC counts remained significantly elevated above baseline throughout the entire 14-day monitoring period without returning to preoperative values. A moderate positive correlation between CRP and WBC was observed only on day 1 (ρ = 0.58, p < 0.05); no significant correlations were found from day 7 onward.

**Conclusion:**

The postoperative kinetics of point-of-care CRP and total WBC counts are fundamentally different after TPLO. CRP provides a reliable, dynamic profile that accurately reflects the resolution of acute inflammation in uncomplicated cases, whereas total WBC count is an unreliable biomarker due to persistent non-specific elevation from surgical stress. These findings support serial point-of-care CRP measurement as a superior objective tool for monitoring postoperative recovery and early detection of complications in dogs undergoing TPLO.

## INTRODUCTION

Surgical intervention, although therapeutic, invariably triggers a cascade of physiological responses proportional to the degree of tissue trauma. Tibial plateau leveling osteotomy (TPLO) is a widely performed and effective surgical procedure in veterinary orthopedics for the treatment of cranial cruciate ligament rupture (CCLr) in dogs [1–3]. Despite its high success rate, TPLO is classified as a major (Level 4) orthopedic procedure because it involves extensive soft tissue dissection, osteotomy, and placement of metallic implants [4]. This degree of surgical trauma induces a well-characterized systemic inflammatory response syndrome, also referred to as the acute phase response (APR) [5]. The APR is mediated by pro-inflammatory cytokines (e.g., IL-1, IL-6, TNF-α), which stimulate hepatic synthesis of acute phase proteins (APPs) [6].

Objective and accurate monitoring of the postoperative inflammatory response is essential for effective clinical management [7]. Such monitoring enables clinicians to distinguish the expected, uncomplicated inflammatory trajectory from postoperative complications, particularly surgical site infections (SSIs) [8]. SSIs remain a significant cause of morbidity in orthopedic procedures, and their reported incidence following TPLO highlights the need for reliable postoperative monitoring tools [9–11]. Traditionally, the total white blood cell (WBC) count derived from a complete blood count (CBC) has been used to assess inflammation and infection [12, 13]. However, its diagnostic utility in the immediate postoperative period is limited. Postoperative leukocytosis is a nonspecific response influenced by factors such as surgical stress, anesthesia, and hospitalization. These factors induce endogenous corticosteroid release, resulting in a “stress leukogram” characterized by neutrophilia, lymphopenia, and eosinopenia, which may obscure the true inflammatory status [14]. Consequently, total WBC count is often a delayed and insensitive indicator of inflammation, limiting its value in monitoring postoperative recovery [15, 16].

Due to these limitations, attention has shifted toward APPs as more dynamic and specific biomarkers of inflammation [17]. In dogs, C-reactive protein (CRP) is the principal positive APP and is considered the gold standard biomarker for systemic inflammation [18]. Unlike WBC count, serum CRP is synthesized directly in response to inflammatory cytokines, primarily IL-6, and has a short half-life [19]. These characteristics confer superior kinetic properties, allowing CRP to increase rapidly following an inflammatory stimulus and decline promptly once the stimulus resolves [19–21]. This predictable kinetic pattern makes serial CRP measurement an ideal tool for objectively tracking both the magnitude and resolution of postoperative inflammation [22]. A steady decline in CRP indicates uncomplicated recovery, whereas persistent elevation or secondary increases strongly suggest complications, such as SSIs, which often precede clinical signs [23].

Although the clinical utility of CRP following TPLO has been reported previously [11, 24], direct comparisons between CRP and WBC kinetic profiles within the same cohort remain limited. Furthermore, routine clinical use of CRP has historically been limited by delays in external laboratory testing. The development of validated point-of-care (POC) immunofluorescence assays now enables rapid, cage-side CRP measurement, facilitating real-time clinical decision-making [25–27].

Although serum CRP has been increasingly recognized as a sensitive biomarker of systemic inflammation in dogs, important knowledge gaps remain regarding its practical role in postoperative monitoring after TPLO. Previous studies have generally focused on documenting postoperative CRP trends or evaluating CRP in relation to infectious complications, but direct longitudinal comparison of CRP with total WBC count within the same TPLO cohort remains limited. This is clinically relevant because the WBC count remains widely used in routine postoperative assessment despite its known susceptibility to nonspecific influences, including surgical stress, anesthesia, corticosteroid release, and hospitalization. As a result, clinicians still lack sufficient evidence on whether serial CRP measurements offer a meaningful advantage over conventional leukogram-based monitoring during the early recovery period after major orthopedic surgery. In addition, although laboratory-based CRP assays have shown diagnostic value, their clinical utility in real-time postoperative decision-making has historically been limited by delayed turnaround time and reduced accessibility in routine practice. The growing availability of validated POC CRP assays presents an opportunity to address this limitation; however, evidence supporting their use for serial monitoring after TPLO remains scarce. Therefore, a more detailed prospective evaluation of the postoperative kinetic behavior of POC CRP in comparison with total WBC count is needed to clarify which biomarker more accurately reflects normal inflammatory resolution after uncomplicated TPLO.

Therefore, the present study was designed to prospectively investigate the postoperative inflammatory kinetics of serum CRP and total WBC count in dogs undergoing TPLO and to directly compare their temporal patterns over a 14-day recovery period. Using a validated POC immunofluorescence assay, this study aimed to determine whether serial CRP measurement could provide a more dynamic, objective, and clinically informative assessment of postoperative inflammatory resolution than total WBC count. In particular, the study sought to characterize the magnitude and timing of postoperative biomarker changes, identify the pattern of return toward baseline in uncomplicated recovery, and assess whether WBC count remains persistently elevated despite apparent inflammatory resolution. By addressing these points, the study determineaimed to establish whether POC CRP monitoring offers superior value as a practical biomarker for routine postoperative surveillance after TPLO and whether it may better assist clinicians in distinguishing expected surgical inflammation from abnormal recovery trajectories.

## MATERIALS AND METHODS

### Ethical approval

Ethical approval for this prospective observational study was obtained before study initiation from the Animal Research Ethics Committee of Mahanakorn University of Technology, Bangkok, Thailand (Protocol No. ACUC-MUT-2025/004). The study was conducted in accordance with institutional guidelines for the ethical use of animals in research and complied with the ARRIVE 2.0 recommendations for transparent reporting of animal studies. Only client-owned dogs diagnosed with unilateral CCLr and scheduled to undergo TPLO as part of routine clinical management at Samut Songkhram Animal Hospital, Samut Songkhram, Thailand, were enrolled. No experimental surgical procedures were performed solely for research purposes, and all perioperative interventions, anesthesia, analgesia, antibiotic prophylaxis, hospitalization, and postoperative monitoring were undertaken in accordance with standard clinical practice and in the best interests of the animals. Written informed consent was obtained from all owners before enrolment, after explanation of the study objectives, the blood sampling schedule, and the use of clinical data for research. Every effort was made to minimize pain, distress, and handling stress during sample collection and postoperative follow-up.

### Study period and location

This investigation was conducted as a prospective observational cohort study from November 2024 to October 2025 at Samut Songkhram Animal Hospital, Samut Songkhram, Thailand.

### Animals and inclusion and exclusion criteria

Thirteen client-owned dogs (n = 13) diagnosed with unilateral CCLr were prospectively enrolled

Inclusion criteria: Dogs were eligible if they had a confirmed diagnosis of unilateral CCLr based on orthopedic examination (including a positive cranial drawer sign) and radiographic findings, and were otherwise systemically healthy without concurrent orthopedic conditions (e.g., hip dysplasia).

Exclusion criteria: Dogs were excluded if they presented with bilateral CCLr, had undergone previous stifle surgery, or had concurrent systemic diseases (e.g., hyperadrenocorticism, diabetes mellitus, hypothyroidism). Dogs with pre-existing infectious conditions, evidence of infection (e.g., elevated CRP >20 mg/L), or those that had received long-acting corticosteroids within 30 days prior to surgery were also excluded to avoid confounding effects on WBC or CRP kinetics.

The study population comprised various breeds (e.g., Thai Ridgeback, Alaskan Malamute, Shiba Inu, Pomeranian, Siberian Husky, Welsh Corgi, Labrador Retriever, Golden Retriever) with a wide weight range (7.65–55.8 kg; mean 29.4 ± 13.5 kg), as detailed in Table 1. This diversity allowed for the assessment of biomarker consistency across different canine phenotypes.

**Table 1:** Clinical history, diagnosis, and surgical techniques of orthopedic procedures performed in 13 dogs.

No.	Breed	Gender	Weight (kg)	Diagnosis	Technique
1	Thai Ridgeback	male	20.4	CCLr	TPLO
2	Thai Ridgeback	female	23.85	CCLr	TPLO
3	Alaskan Malamute	male	27.2	CCLr	TPLO
4	Thai Ridgeback	male	31.8	CCLr	TPLO
5	Shiba Inu	male	9.7	CCLr	TPLO
6	Pomeranian	male	7.65	CCLr	TPLO
7	Siberian Husky	female	32.0	CCLr	TPLO
8	Alaskan Malamute	male	55.8	CCLr	TPLO
9	Thai Ridgeback	male	30.0	CCLr	TPLO
10	Welsh Corgi	male	15.2	CCLr	TPLO
11	Siberian Husky	female	32.0	CCLr	TPLO
12	Labrador Retriever	male	44.5	CCLr	TPLO
13	Golden Retriever	female	32.0	CCLr	TPLO

CCLr = Cranial cruciate ligament rupture, TPLO = Tibial plateau leveling osteotomy.

### Surgical procedure and perioperative management

Surgical technique: All dogs underwent TPLO, performed by the same surgical team to ensure technical consistency. The procedure followed standardized techniques, including a medial approach to the proximal tibia, radial osteotomy using a dedicated oscillating saw, and rotation of the tibial plateau to a predetermined target angle. Bone segments were stabilized using a TPLO-specific locking plate and screws. Intraoperative meniscal evaluation and release were performed as indicated during initial joint exploration. The total surgical duration ranged from 120 to 180 min, corresponding to a Level 4 major orthopedic procedure according to the five-level severity classification system (Table 2).

**Table 2:** A five-level severity classification for veterinary orthopedic procedures.

Level	Duration	Level of bone manipulation	Example
1	<30 min	Minimal/None	Removal of minor implant
2	30–60 min	Drilling holes, diagnostic arthroscopy	Diagnostic arthroscopy of the stifle, pinning a phalanx
3	1–2 h	Osteotomy or simple plating	Fixation with plate and screws, medial patellar luxation repair with trochleoplasty
4	2–3 h	Complex and lengthy surgery	Tibial plateau leveling osteotomy, total hip replacement
5	>3 h	Complex osteotomy and extensive fixation	Complex pelvic or acetabular fracture repair, revision total hip replacement

Anesthesia and antibiotic prophylaxis: A standardized anesthetic protocol was applied. Premedication was administered to ensure sedation and preemptive analgesia. Anesthesia was induced with propofol (4 mg/kg IV) and maintained with isoflurane in 100% oxygen. Prophylactic cefazolin (22 mg/kg IV) was administered 30 min prior to the first incision and repeated every 90 min throughout the procedure. Intraoperative fluid therapy was maintained at 5 mL/kg/h.

Perioperative analgesia and postoperative care: To manage surgical pain and standardize physiological stress, a multimodal analgesic protocol was employed, including intraoperative opioid administration followed by postoperative buprenorphine and non-steroidal anti-inflammatory drugs. All dogs were hospitalized for at least 3 days postoperatively for professional monitoring. Clinical assessments, including standardized pain scoring and surgical site inspection, were performed daily to ensure uncomplicated recovery prior to discharge.

### Sample collection

Venous blood samples (approximately 3 mL per collection) were obtained from the cephalic vein at seven predefined time points: immediately before surgery (Day 0) and on postoperative days (dpo) 1, 3, 5, 7, 10, and 14. To minimize diurnal variation in biomarker concentrations, all samples were collected within a consistent time window between 08:00 and 10:00. Prior to each collection, dogs were fasted for 12 h, and minimal restraint was used to reduce physiological stress. Immediately after collection, blood samples were divided into EDTA tubes (1 mL) for CBC analysis and serum separator tubes (2 mL) for CRP measurement. Serum was separated by centrifugation at 1,500 × *g* for 10 min and analyzed immediately.

### Biomarker analysis and assay validation

Serum CRP concentrations were quantified using a validated POC immunofluorescence assay (SmartDiag IFA Canine CRP, Vet Planet Co., Ltd., Bangkok, Thailand). The SmartDiag® assay has been validated for canine serum, demonstrating intra-assay coefficients of variation (CV) <5% and inter-assay CV <10%. The reference interval for healthy dogs applied in this study was <10 mg/L, with a validated measuring range of 2–250 mg/L. All reagents were equilibrated to 20°C–25°C, and the analyzer was calibrated using the manufacturer-provided internal control card for each batch to ensure analytical precision. Subsequent CRP measurements used SmartDiag® reagents.

Concurrent total WBC counts were measured using a calibrated automated hematology analyzer (ProCyte Dx, IDEXX Laboratories, Inc., Westbrook, ME, USA). The reference interval applied was 6–17 × 10³/μL. Differential leukocyte counts were not analyzed, as the focus was on total WBC kinetics for direct comparison with serum CRP.

All analyses were performed by trained technicians. CRP assays included daily calibration and quality controls to ensure analytical consistency.

### Statistical analysis

An a priori power analysis based on preliminary pilot data determined that a sample size of 13 dogs would provide 80% power (α = 0.05) to detect a 50% increase in CRP concentration on postoperative day 1. Data distribution was assessed for normality using the Shapiro–Wilk test. All variables at each time point were normally distributed (p > 0.05).

Changes in CRP and WBC concentrations across the seven predefined time points were analyzed using repeated-measures one-way analysis of variance. To control for Type I error due to multiple comparisons, Dunnett’s post-hoc test was used to compare each postoperative time point with the preoperative baseline (Day 0). All statistical analyses were performed using GraphPad Prism version 9.0 (GraphPad Software, San Diego, CA, USA). No adjustments for missing data were necessary, as all 13 dogs completed the 14-day study protocol. Statistical significance was set at p < 0.05.

## RESULTS

### Study population

The study included 13 client-owned dogs diagnosed with CCLr. All dogs subsequently underwent TPLO surgery. According to the standardized five-level orthopedic severity classification described by Sumalai *et al*. [28], TPLO is categorized as a Level 4 (high-severity) procedure (Table 2).

### Preoperative status

At baseline (Day 0), the mean serum CRP concentration was 19.26 ± 18.26 mg/L (Table 3). Although the reference interval for healthy dogs was <10 mg/L, several individuals exhibited mild preoperative elevations (range, 11.40–75.60 mg/L).

**Table 3:** Serial mean concentrations, standard deviation (SD), and clinical interpretation of serum CRP and total WBC counts in 13 dogs.

Time point	CRP (mg/L) (Mean ± SD)	CRP range (Min–Max)	WBC (× 10³/μL) (Mean ± SD)	WBC range (Min–Max)	Clinical interpretation
0 dpo	19.26 ± 18.26	11.40–75.60	11.77 ± 4.44	6.15–19.85	Mild elevation due to chronic CCLr
1 dpo	59.51 ± 20.61*	14.90–87.10	16.97 ± 4.52*	7.86–26.84	Significant acute phase response peak
3 dpo	41.27 ± 21.31*	15.90–71.10	16.02 ± 4.81*	7.08–27.12	–
5 dpo	23.02 ± 17.91*	12.10–74.30	14.75 ± 5.18*	7.91–26.40	–
7 dpo	20.11 ± 20.73	11.10–87.30	16.31 ± 5.30*	8.07–20.65	Divergence: CRP resolved; WBC stayed high
10 dpo	19.35 ± 18.07	11.00–75.90	16.30 ± 6.60*	9.37–29.99	Divergence: CRP resolved; WBC stayed high
14 dpo	21.84 ± 22.65	11.90–85.20	16.65 ± 10.08*	6.58–37.33	Divergence: CRP resolved; WBC stayed high

p < 0.05 compared with 0 dpo (baseline) by repeated-measures one-way analysis of variance. WBC unit corrected to × 10³/μL for standard hematology reporting. CCLr = Cranial cruciate ligament rupture, CRP = C-reactive protein, WBC = White blood cells

### Postoperative CRP kinetics

**Figure 1:**
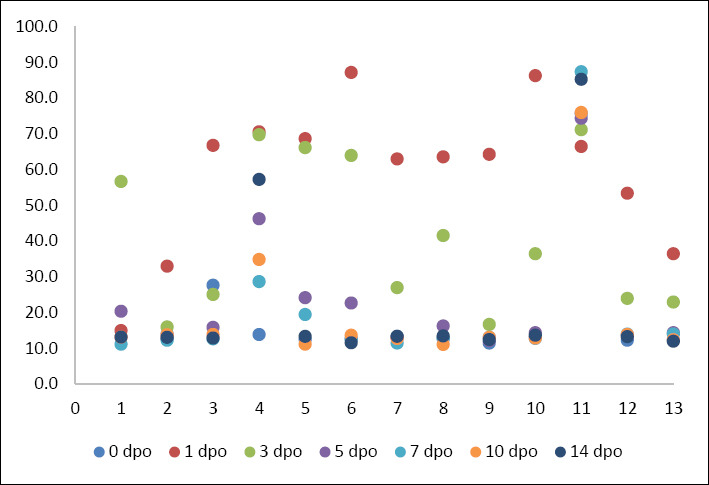
The scatter plot shows serum C-reactive protein concentrations (mg/L) in 13 dogs undergoing tibial plateau leveling osteotomy, measured at 7 time points: pre-operation (0 day postoperative) and 1, 3, 5, 7, 10, and 14 days postoperative.

**Figure 2:**
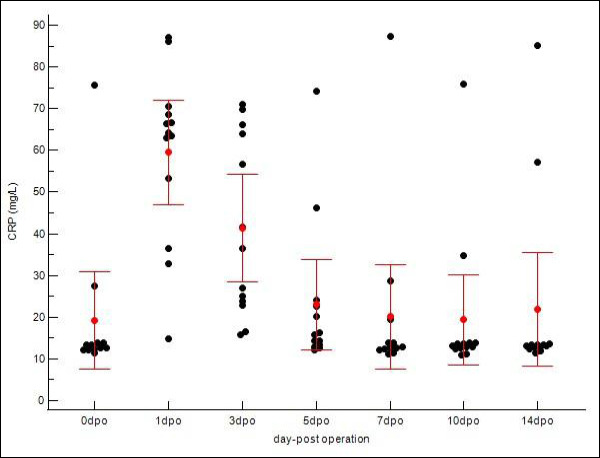
Postoperative kinetics of serum C-reactive protein (CRP) in 13 dogs undergoing tibial plateau leveling osteotomy (TPLO). The scatter dot plot presents individual CRP concentrations at pre-operative (Day 0) and postoperative days 1, 3, 5, 7, 10, and 14. A significant rise in CRP was observed, with mean concentrations peaking at day 1 postoperatively (p < 0.05) and subsequently declining toward baseline, consistent with the expected acute inflammatory response following TPLO.

Mean serum CRP concentration increased significantly from a baseline of 19.26 ± 18.26 mg/L (range, 11.40–75.60 mg/L) to a peak of 59.51 ± 20.61 mg/L (range, 14.90–87.10 mg/L) on postoperative day 1 (p < 0.05). By postoperative day 5, CRP concentrations had decreased markedly to 23.02 ± 17.91 mg/L and approached baseline values by postoperative day 7 (20.11 ± 20.73 mg/L) (Table 3). Individual variability in CRP response is illustrated in Figure 1. The overall kinetic trend is summarized in Figure 2.

### Postoperative WBC kinetics

**Figure 3:**
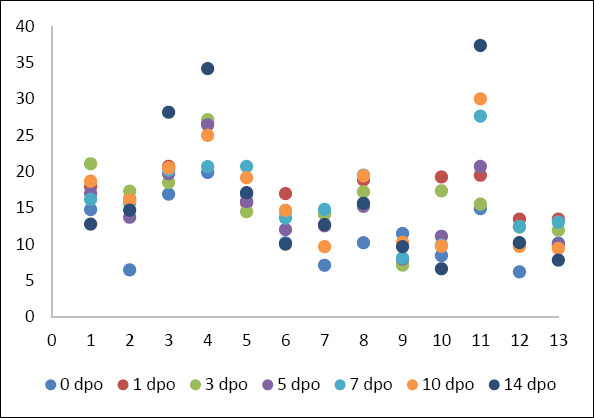
The scatter plot shows white blood cell concentrations (×10³ cells/μL) in 13 dogs undergoing tibial plateau leveling osteotomy, measured at 7 time points: pre-operation (0 day postoperative) and 1, 3, 5, 7, 10, and 14 days postoperative.

**Figure 4:**
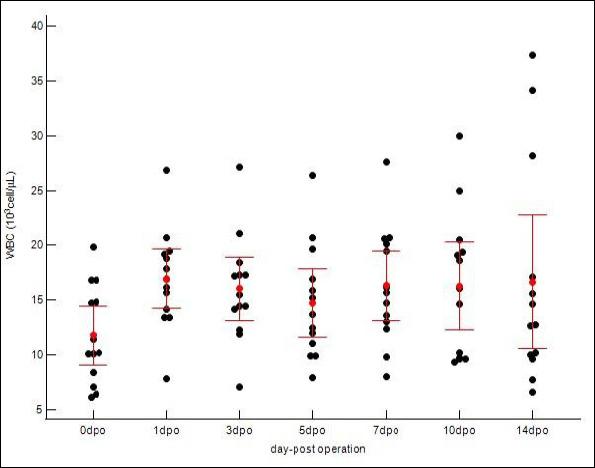
Postoperative kinetics of white blood cells (WBCs) in 13 dogs undergoing tibial plateau leveling osteotomy. The scatter dot plot presents individual WBC counts at pre-operative (Day 0) and postoperative days 1, 3, 5, 7, 10, and 14. A significant increase in WBC counts was observed, with mean values peaking on postoperative day 1 (p < 0.05) and remaining steady until day 14, consistent with the expected acute inflammatory response following tibial plateau leveling osteotomy.

Total WBC counts increased from a baseline of 11.77 ± 4.44 × 10³/μL (range, 6.15–19.85 × 10³/μL) to a peak of 16.97 ± 4.52 × 10³/μL (range, 7.86–26.84 × 10³/μL) on postoperative day 1 (p < 0.05). In contrast to CRP, WBC counts remained elevated above baseline throughout the observation period, with a mean of 16.65 ± 10.08 × 10³/μL (range, 6.58–37.33 × 10³/μL) even at postoperative day 14 (Table 3). Individual scatter data for WBC counts are shown in Figure 3. The persistent kinetic profile is displayed in Figure 4.

### Individual variability and summary statistics

The individual variability and summary statistics for both biomarkers are detailed in Table 3. Although mean values demonstrated a clear kinetic trend, individual scatter plots (Figures 1 and 3) revealed variability in response magnitude. Notably, despite individual differences in peak concentrations on postoperative day 1, all dogs exhibited the same directional pattern: a sharp increase followed by progressive decline in CRP, whereas WBC counts remained persistently elevated without returning to baseline. No significant outliers were identified that deviated from these primary kinetic patterns.

To assess the relationship between the two biomarkers, Spearman’s rank correlation coefficient (ρ) was calculated at each time point. A moderate positive correlation was observed only on postoperative day 1 (ρ = 0.58, p < 0.05), when both markers reflected the acute response to surgical trauma. However, from postoperative day 7 through postoperative day 14, no significant correlations were detected (p > 0.05), supporting the divergent kinetic profiles in which CRP concentrations returned toward baseline while WBC counts remained nonspecifically elevated.

## DISCUSSION

### Postoperative inflammatory response after TPLO

This study characterized the postoperative kinetics of serum CRP and total WBC counts in dogs undergoing TPLO, a Level 4 (high-severity) orthopedic procedure. The results demonstrated that both biomarkers increased significantly within 24 h after surgery, confirming activation of the acute inflammatory response induced by TPLO. However, their subsequent kinetic profiles differed considerably. Serum CRP concentrations declined progressively after the postoperative peak, whereas total WBC counts remained elevated throughout the study period. These findings indicate that CRP more accurately reflects the resolution of postoperative inflammation and may therefore be a more reliable biomarker for monitoring recovery after major orthopedic surgery.

The observed preoperative elevation in CRP in some dogs was likely due to chronic low-grade systemic inflammation secondary to CCLr. Although several dogs had mildly increased baseline CRP concentrations, the marked increase observed on postoperative day 1 represented a consistent and significant response relative to each dog’s individual baseline. This finding supports the interpretation that the postoperative rise in CRP was attributable primarily to surgical trauma rather than pre-existing variation alone.

### Kinetic profile of CRP after surgery

The CRP pattern observed in this study, characterized by a sharp peak at 1 dpo followed by a gradual decline, is consistent with the well-established profile of the APR after major surgical trauma. This 24-h postoperative peak agrees with previous reports in canine orthopedic surgery [29–31] and is consistent with findings specifically reported for the TPLO by Löfqvist et al. [11]. By 14 dpo, CRP concentrations had returned toward baseline values, which is consistent with an uncomplicated postoperative course [22].

This predictable pattern strengthens the value of CRP as a postoperative monitoring biomarker [24]. In routine clinical practice, a progressively declining CRP trajectory supports normal recovery, whereas deviations from this expected course, including failure to decline after 48–72 h or a secondary rise, may indicate postoperative complications such as SSI [11, 23]. Therefore, serial CRP measurements can provide objective support for early clinical decision-making and may help identify abnormal recovery before overt clinical deterioration becomes evident.

### Comparison between CRP and WBC kinetics

A major finding of this study was a clear discrepancy between CRP and WBC kinetics, underscoring their distinct diagnostic value in the postoperative setting. Although both biomarkers peaked on postoperative day 1, total WBC counts did not show a comparable decline and remained above baseline throughout the 14-day observation period. This persistent elevation suggests that WBC count is less useful than CRP for monitoring the resolution of acute postoperative inflammation.

This finding is supported by previous studies indicating that total WBC count is a relatively insensitive and nonspecific marker in the early postoperative period. Leukocytosis may reflect a generalized physiological stress response associated with surgery, anesthesia, hospitalization, and corticosteroid release rather than ongoing inflammatory activity alone [15, 32, 33]. Consequently, reliance on total WBC counts alone may lead to inaccurate interpretation of postoperative status. In contrast, serum CRP, because of its direct induction by inflammatory cytokines and shorter half-life, provides a more dynamic and clinically relevant measure of active inflammation and its resolution [18–21].

### Clinical relevance of POC CRP monitoring

The consistency of the CRP decline across the cohort, despite variation in breed and body weight, supports the practical clinical value of POC CRP measurement. All dogs showed a reduction in CRP concentrations by postoperative day 7 relative to their day-1 peak, reinforcing the reliability of this marker in identifying expected postoperative recovery in uncomplicated cases.

The use of a validated in-clinic POC assay further enhances the applicability of CRP monitoring in daily veterinary practice. Historically, the clinical use of CRP was limited by delays associated with external laboratory testing. In contrast, rapid cage-side CRP testing enables real-time assessment of inflammatory status and may facilitate earlier intervention when abnormal postoperative trends are detected. This is especially important after major orthopedic procedures such as TPLO, in which early differentiation between expected inflammation and emerging complications remains clinically challenging.

### Relationship with surgical severity

The marked inflammatory response observed in both biomarkers at 1 dpo is consistent with the classification of TPLO as a Level 4 orthopedic procedure. TPLO involves osteotomy, considerable soft-tissue manipulation, and implantation of metallic hardware, all of which contribute substantially to APR activation [9–11]. The magnitude of the CRP peak may therefore reflect the degree of surgical trauma, supporting previous reports that more invasive procedures induce stronger acute phase responses [34].

In this context, the present findings further support the concept that postoperative CRP kinetics are closely linked to tissue trauma and subsequent inflammatory resolution. Thus, CRP may serve not only as a biomarker of recovery but also as an objective index of the biological impact of surgical intervention.

### Limitations of the study

Several limitations should be considered when interpreting these findings. First, the sample size was relatively small (n = 13), which may limit statistical power and reduce the generalizability of the results. Second, because all enrolled dogs underwent the same orthopedic procedure, comparisons across different levels of surgical severity were not possible. Third, the study focused only on CRP and total WBC counts. Inclusion of additional APPs, such as serum amyloid A (SAA) or haptoglobin, might have provided a broader characterization of the APR [11]. Finally, all dogs in this cohort experienced uncomplicated recovery. Inclusion of dogs with confirmed postoperative SSI or other inflammatory complications would be required to determine clinically useful diagnostic cutoff values and strengthen the translational value of the findings.

### Overall interpretation

Taken together, the results indicate that CRP and WBC count follow fundamentally different postoperative trajectories after TPLO. Although both markers respond to surgical trauma, CRP more closely mirrors the expected course of inflammatory resolution, whereas WBC count remains nonspecifically elevated for a longer period. This difference highlights the superiority of CRP over total WBC count for postoperative monitoring in dogs undergoing major orthopedic surgery. Accordingly, serial POC CRP measurement appears to be a practical and objective approach for assessing postoperative recovery and identifying deviations from the expected healing trajectory.

## CONCLUSION

The present study demonstrated that both serum CRP and total WBC count increased significantly within 24 h following TPLO, reflecting the expected acute inflammatory response associated with a Level 4 orthopedic procedure. However, their postoperative kinetic profiles differed markedly. Serum CRP showed a predictable, progressive decline after peaking at 1 dpo, approaching baseline by 7 dpo, consistent with the normal resolution of inflammation in uncomplicated recovery. In contrast, total WBC counts remained persistently elevated throughout the 14-day observation period, indicating a nonspecific response likely influenced by physiological stress rather than true inflammatory activity.

From a clinical perspective, these findings highlight the superior utility of CRP as a biomarker for postoperative monitoring. Serial measurement of CRP provides a dynamic and objective assessment of inflammatory status, enabling clinicians to distinguish between expected postoperative recovery and potential complications such as SSIs. The use of a validated POC CRP assay further enhances its practical applicability by allowing rapid, real-time, cage-side evaluation, thereby supporting timely clinical decision-making and potentially improving patient outcomes in veterinary orthopedic practice.

A key strength of this study lies in its prospective design and standardized surgical and perioperative protocols, which minimized variability and allowed direct comparison of biomarker kinetics within the same cohort. Additionally, serial measurements at multiple postoperative time points provided a comprehensive characterization of inflammatory trends. The consistency of CRP responses across diverse breeds and body weights further supports the robustness and generalizability of the findings within the context of uncomplicated TPLO recovery.

Nevertheless, future studies are warranted to expand upon these findings. Larger sample sizes and inclusion of dogs with confirmed postoperative complications, particularly SSIs, would enable the establishment of clinically relevant diagnostic thresholds and enhance translational applicability. Furthermore, incorporation of additional APP, such as serum amyloid A or haptoglobin, may provide a more comprehensive understanding of the APR and its diagnostic potential. Comparative evaluation across different surgical procedures and severity levels would also help clarify the broader applicability of CRP monitoring in veterinary surgery.

In conclusion, serum CRP exhibits a reliable and clinically meaningful kinetic profile that closely reflects postoperative inflammatory resolution, whereas total WBC count remains a nonspecific and less informative marker. Serial POC CRP measurements represent a valuable, objective, and practical tool for postoperative monitoring in dogs undergoing TPLO and have the potential to improve early detection of complications and optimize clinical management.

## DATA AVAILABILITY

The supplementary data can be made available from the corresponding author upon request.

## AUTHORS’ CONTRIBUTIONS

TS, SR, SW, RL, and TT: Conceptualization, methodology, formal analysis. TS and SR: Funding acquisition and investigation. TS, SR, and SW: Project administration. SR: Supervision. TS, SR, and SW: Drafted and revised the manuscript. All authors have read and approved the final version of the manuscript.
